# European expert consensus recommendations on the primary care use of direct oral anticoagulants in patients with venous thromboembolism

**DOI:** 10.1186/s12875-024-02314-7

**Published:** 2024-03-18

**Authors:** Carter Patrice, Fuat Ahmet, Haas Sylvia, Smyth Elizabeth, Brotons Carlos, Cools Frank, Bauersachs Rupert, Hobbs F. D. Richard

**Affiliations:** 1 Health Economics & Outcomes Research Ltd, Rhymney House, Unit A Copse Walk, Cardiff Gate Business Park, Cardiff, CF23 8RB UK; 2 School of Health, University of Durham, Durham, DH1, 3LE UK; 3 Primary Care, Durham University, Durham, UK; 4 Formerly Technical University of Munich, Munich, Germany; 5 Cambridge University Hospitals NHS Foundation Trust, Cambridge, UK; 6 Sardenya Primary Health Care Centre-Institut de Recerca Sant Pau, Barcelona, Spain; 7 AZ Klina, Department of Cardiology, Augustijnslei 100, Brasschaat, 2930 Belgium; 8 Center of Vascular Research - VASC, Munich, Germany; 9 Cardioangiologic Center, Bethanien CCB, Frankfurt, Germany; 10 Nuffield Department of Primary Care Health Sciences, Radcliffe Observatory Quarter, University of Oxford, Radcliffe Primary Care Building, Oxford, OX2 6GG UK

**Keywords:** Venous thromboembolism (VTE), Direct oral anticoagulants (DOACs), Cancer associated thromboembolism, Primary Care, Formal consensus

## Abstract

**Background:**

Direct oral anticoagulants for the treatment of venous thromboembolism are supported by robust clinical trial evidence. Despite published guidance, general practitioners are faced with increasingly complex decisions and implementation remains sub-optimal in certain real-world scenarios.

**Methods:**

A two stage formal consensus exercise was performed to formulate consensus statements and a summary guide, facilitating optimal management of direct oral anticoagulants in venous thromboembolism patients by generalist physicians across Europe. An online questionnaire distributed to a broad panel (Phase 1), followed by a virtual panel discussion by an expert group (Phase 2) were conducted. Phase 1 statements covered nine management domains, and were developed via a literature review and expert steering committee. Participants rated statements by their level of agreement. Phase 1 responses were collated and analysed prior to discussion and iterative refinement in Phase 2.

**Results:**

In total 56 participants from across Europe responded to Phase 1. The majority had experience working as general practitioners. Consensus indicated that direct oral anticoagulants are the treatment of choice for managing patients with venous thromboembolism, at initiation and for extended treatment, with a review at three to six months to re-assess treatment effect and risk profile. Direct oral anticoagulant choice should be based on individual patient factors and include shared treatment choice between clinicians and patients; the only sub-group of patients requiring specific guidance are those with cancer.

**Conclusion:**

Results demonstrate an appreciation of best practices, but highlight challenges in clinical practice. The patient pathway and consensus recommendations provided, aim to highlight key considerations for general practice decision making, and aid optimal venous thromboembolism treatment.

**Supplementary Information:**

The online version contains supplementary material available at 10.1186/s12875-024-02314-7.

## Background

Venous thromboembolism (VTE), which includes deep vein thrombosis (DVT) and pulmonary embolism (PE), refers to the formation of a clot which fully or partially obstructs blood flow [1]. VTE is the third most common cause of vascular mortality worldwide after myocardial infarction and stroke [2]. Approximately half of VTE events are unprovoked, while others are linked to known risk factors including surgery, acute admission to hospital, malignancy, older age, prolonged bedbound, prior use of the combined oral contraception pill, inherited and acquired types of thrombophilia, hormone-replacement therapy and pregnancy [3]. 

Historically, vitamin K antagonists (VKAs) were the standard care for VTE therapy; however, robust clinical trial evidence supporting the use of direct oral anticoagulants (DOACs), which include apixaban, dabigatran, edoxaban and rivaroxaban has resulted in their widespread implementation across clinical practice [4]. Furthermore, comprehensive published clinical guidance from across Europe now consistently supports the use of DOACs for both the treatment and prevention of VTE [5–10]. 

Despite these consistent guideline recommendations, some of the recommendations within the guidelines are based on low level evidence, and uncertainty remains regarding management in some clinical scenarios experienced in real-world practice [8, 11]. With the expanding role of DOACs, uncertainty stated in guidance [8], and some evidence that available guidance is not always implemented [12], generalist physicians are faced with increasingly complex decisions relating to appropriate agents and duration of treatment for a diverse population of individual patients. Therefore, there remains an educational need to formulate recommendations based on clinical experience to act as a practical management reference tool.

### Objective

To formulate evidence-based expert consensus statements and a summary guide to facilitate optimal management of VTE patients with anticoagulation by DOACs by generalist physicians across Europe.

## Methods

To develop recommendations for practice we conducted a formal consensus exercise, based on the Delphi technique, a structured approach where expert opinion is elicited to enable consolidation of opinions into single statements [13].

The formal consensus exercise was performed in two stages; Phase 1, an online questionnaire which encompassed 53 statements/questions, divided into the following sections: participant information and introductory statements, current landscape and need for a guideline, DOACs versus other anticoagulants, treatment initiation, duration of treatment, and treatment within specific patient groups. The statements were developed following a targeted literature search to identify existing, international published guidelines, articles, commentaries, and grey literature providing guidance on the clinical management of DOACs in patients with VTE. Using the publications identified (listed in Additional file 1), initial statements were drafted, these underwent several rounds of revision with input from an expert, multidisciplinary steering committee (FDRH, AF, SH, ES).

The Phase 1 questionnaire was distributed using the online ‘SmartSurvey’ (https://www.smartsurvey.co.uk/) platform. A hyperlink was distributed via email to a broad panel of health care professionals with an interest in cardiovascular disease in primary care. Participants were identified from relevant publications via targeted literature searching. Additionally, the board members of the European Primary Care Cardiovascular Society circulated the survey invite to up to five county level members of their relevant associations, and members of the Primary Care Cardiovascular Society (PCCS) who opted to receive direct pharma emails were invited to participate. Individuals did not receive any renumeration for their participation in the survey.

At the beginning of the survey all participants gave informed consent to participate, and were made aware they could withdraw their responses at any time. To be considered eligible, participants were required to be healthcare practitioners in Europe, with an interest in primary care treatment of people with VTE.

Participants were asked to rate the statements using a 7-point Likert scale relevant to their level of agreement with the statement. Participants were encouraged to leave comments to enable the statements to be redrafted if consensus was not reached. There was also an option for participants to indicate they did not understand the statement or had insufficient knowledge to provide a rating.

Phase 2 was a virtual consensus meeting, where the results from Phase 1 were considered by a group of experts, representing several European countries (FDRH, AF, SH, ES, FC, RB and CB). Phase 2 deliberately involved a smaller panel than Phase 1, to ensure focussed group discussion. The members of the expert panel included the multidisciplinary steering committee (FDRH, AF, SH, ES) and three members who all participated in Phase 1 (FC, RB and CB), selected according to their expertise across clinical fields. FDRH was initially approached as the Chair of the European Primary Care Cardiovascular Society, and further participants were suggested or recommended by discussion between FDRH and PC.

Responses from Phase 1 were consolidated and analysed using descriptive statistics to summarise findings. For data analysis purposes, ratings were grouped into three categories: 1–3 (disagree), 4 (neither agree nor disagree) or 5–7 (agree). Statements with 80% or greater agreement were considered to show strong consensus, and statements with 60–79% agreement were considered to have some consensus. Statements with less than 60% agreement were considered to have poor/no consensus.

During Phase 2, the collated statements from Phase 1 were presented, discussed, and refined into final consensus statements during a virtual consensus meeting. The panellists in Phase 2 were presented with the results for each topic and they exchanged opinions on the statements and the levels of consensus calculated from Phase 1; panellists considered the written comments provided by participants during Phase 1, and if required, refined statements in line with their agreed group opinion, and the comments provided by Phase 1 participants. Assumptions were refined by a facilitator (PC) who captured decisions and presented these back for final validation and approval.

## Results

**Fig. 1:**
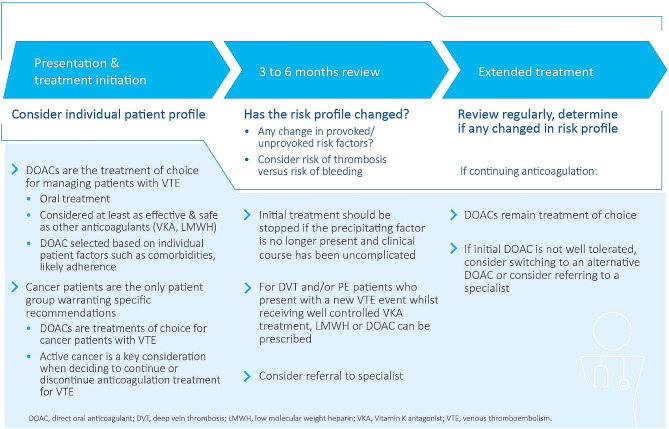
Patient pathway to support generalist physicians in management of VTE

The complete results from Phase 1 are presented in the supplementary material (Additional file 2), the overall recommendations are summarized as a patient pathway in Fig. 1. (*Patient pathway to support generalist physicians in management of VTE*), and a combined narrative synthesis of findings from Phase 1 and Phase 2 are presented below.

### Phase 1 participant characteristics

A total of 56 participants either fully completed (*n* = 40) or partially completed (*n* = 16) the online questionnaire (Phase 1). The majority of respondents were from key European countries including Belgium (*n* = 14), the UK (*n* = 12), Italy (*n* = 10), Germany (*n* = 6), Spain (*n* = 5), other contributors were from Poland (*n* = 2), Greece (*n* = 2), Russia (*n* = 2), Romania (*n* = 1), Israel (*n* = 1) and the United Arab Emirates (*n* = 1).

When stating in which clinical setting they worked, participants classed themselves as general practitioners (*n* = 29), primary care specialists (*n* = 11), working within specialist centers (*n* = 16), or working within the secondary care setting (*n* = 6), some participants selected multiple settings.

Within the primary care setting, the median number of patients managed per annum was reported to be 2,000 (range: 100 − 15,000); the estimated proportion of these patients who had VTE was 4% (median; range 1-100%) and the proportion of VTE patients that were prescribed DOACs was 80% (median; range: 0-100%). The median number of patients managed per annum was similar for participants in the secondary care setting (median: 2,250; range: 500 − 15,000); however, a higher proportion of these patients were reported to have VTE (median: 35%, range:1-100%) and were prescribed DOACs for VTE treatment (median: 85%, range: 50–99%). The numbers managed by participants were self-declared in the first section of the questionnaire, which requested background information on the Phase 1 participants.

### Current landscape and requirement for consensus recommendations

#### Key recommendation

- *Concise and simple evidence-based recommendations for the management of VTE patients in primary care are needed to aid optimization of care*.

**Fig. 2:**
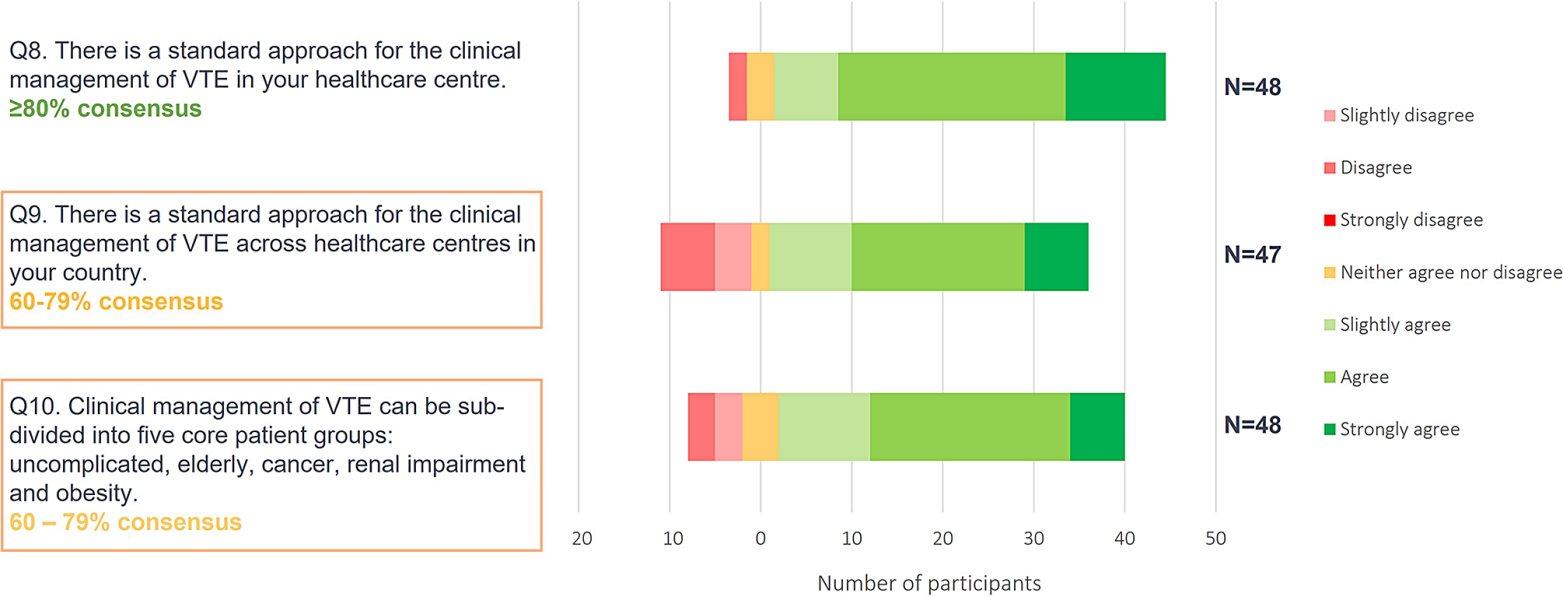
Phase 1 online questionnaire consensus ratings on the current VTE treatment landscape

Phase 1 results demonstrated that participants consider VTE to be consistently managed within their respective treatment centres, but not across their representative countries; findings were consistent across the varying geographical locations of participants (Fig. 2. *Phase 1 online questionnaire consensus ratings on the current VTE treatment landscape*). Phase 2 panellists confirmed that the management of VTE can deviate substantially across primary care centres, again this variation in practice was considered true across panellists representing different European countries. The results support the need for simple, accessible recommendations on VTE management for generalist physicians to aid consistent management.

### DOACs versus other anticoagulants

#### Key recommendations

- *DOACs are the treatment of choice for managing patients with VTE.*
- *DOACs offer an oral treatment that is at least as effective and safe as other anticoagulants (VKA and LMWH).*
- *DOAC choice should be based on individual patient factors, such as comorbidities and likely adherence.*

**Fig. 3:**
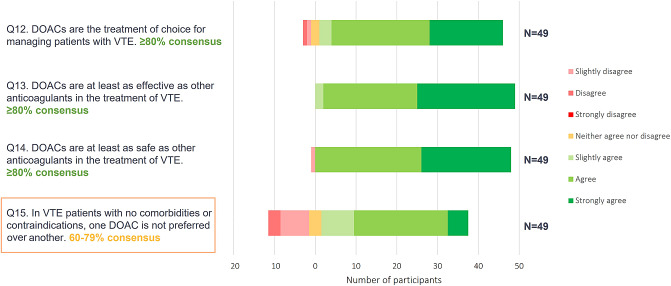
Phase 1 online questionnaire consensus ratings for DOACs versus other anticoagulants

**Fig. 4:**
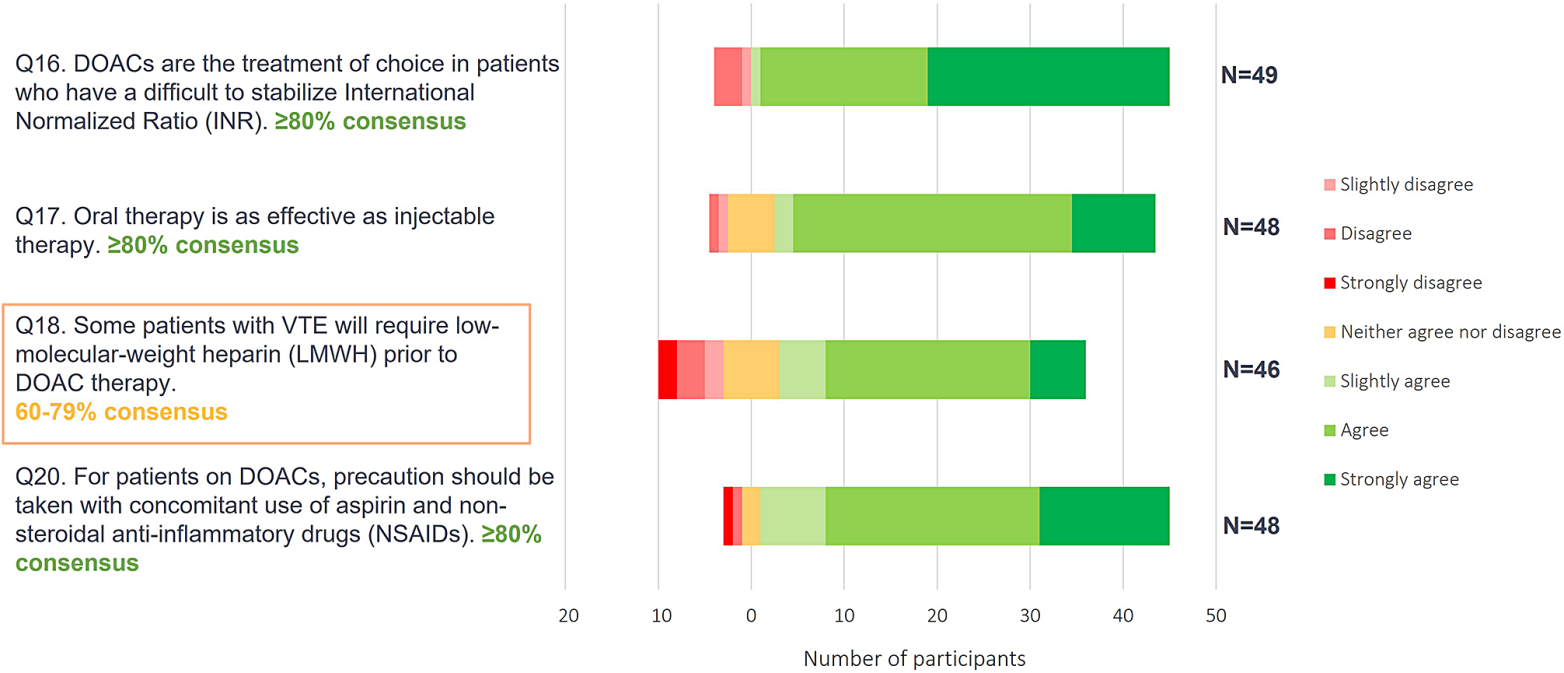
Phase 1 online questionnaire consensus ratings for DOACs versus other anticoagulants

Results from Phase 1 showed a high level of agreement with the statements presented regarding DOAC use versus other anticoagulants in the treatment of VTE (Fig. 3. *Phase 1 online questionnaire consensus ratings for DOACs versus other anticoagulants* and Fig. 4. *Phase 1 online questionnaire consensus ratings for DOACs versus other anticoagulants*).

The Phase 2 panellists supported the views expressed in Phase 1, amending the initial statements and key messages minimally. Panellists in Phase 2 highlighted the fact that Phase 1 results provided evidence that no one DOAC should be recommended over another. It was acknowledged that, in practice, apixaban and rivaroxaban may be more widely prescribed as they do not require pre-treatment with a parenteral anticoagulant, for example with low molecular weight heparin (LMWH); however, if an alternative DOAC is considered more suitable, and pre-treatment with LMWH is required, this should not exclude its use. Phase 2 discussions also highlighted the importance of distinguishing between DVT and PE, and that raising awareness of PE is important within primary care. Phase 2 panellists noted that PE can present with varying symptoms and is not as easily diagnosed as DVT. PE and DVT require different management pathways, and those with PE should be referred to specialist care. The panel recognized that individualized treatment decision-making for DOAC of choice is important, and that a summary table of DOAC characteristics (i.e., dosing schedule, requirement of LMWH run-in, patient risk factors) to aid treatment choice may be valuable (Table 1).

**Table 1:** Characteristics of DOACs for patients with VTE, and considerations relating to patient risk factors/comorbidities

DOAC		Dosing schedule	Requirement of LMWH pre-treatment	Considerations for patients with renal impairment	Considerations for patients in relation to body weight	Considerations for patients who are elderly
Apixaban (*Eliquis*)	Initial treatment of DVT or PE	First 7 days: 10 mg twice daily	Not required	CrCl – 15 to 29mL/min – use with caution CrCl < 15mL/min – not recommended for use	None	None
		Followed by: 5 mg twice daily (for 3–6 months)				
	Continuation of treatment if prevention of recurrence is required	2. 5 mg twice daily				
Rivaroxaban (*Xarelto*)	Initial treatment of DVT or PE	Day 1 to day 21: 15 mg twice daily	Not required	CrCl – 30–49mL/min – patients should be treated with 15 mg twice daily for the first 3 weeks, when recommended dose is 20 mg, a reduction to 15 mg should be considered CrCl – 15 to 29mL/min – to be used with caution in these patients. Patients should be treated with 15 mg twice daily for the first 3 weeks, when recommended dose is 20 mg, a reduction to 15 mg should be considered. Use according to individual assessment of thromboembolic risk and risk of bleeding CrCl < 15mL/min – not recommended for use	None	None
		Day 22 onwards: 20 mg once daily				
	Continuation of treatment if prevention of recurrence is required	10 mg once daily or 20 mg once daily (in case of patients at high risk)				
Edoxaban (*Lixiana*)	Initial treatment and continuation of treatment for DVT and PE	60 mg once daily following at least 5 days of parental anticoagulation	Required	CrCl – 15 to 30mL/min – use 30 mg once daily	For those with body weight ≤ 60 kg use 30 mg once daily	None
Dabigatran (*Pradaxa*)	Treatment and continuation of treatment for DVT and PE	150 mg twice daily following at least 5 days of treatment with a parenteral anticoagulant	Required	CrCl < 30 mL/min dabigatran is contraindicated CrCl 30 mL/min to 50 mL/min use daily dose of 300 mg or 220 mg according to individual assessment of thromboembolic risk and risk of bleeding	None	For those aged ≥ 80 years twice daily dose of 110 mg For those aged 75–80 years use daily dose of 300 mg or 220 mg according to individual assessment of thromboembolic risk and risk of bleeding

*Summarised information within this table is from the Electronic Medicines Compendium (EMC), please refer to the website for more detailed information* ^*23*^

### Consideration for specific patient populations

#### Key recommendations

- *The only sub-group of patients who warrant their own specific recommendations on VTE treatment, are those with cancer.*
  - *DOACs are the treatment of choice for cancer patients with VTE*
  - *Active cancer is a key consideration when deciding to continue or discontinue anticoagulation treatment for VTE*

Phase 1 comprised a number of statements relating to specific VTE patient subgroups, including patients with cancer, the elderly, patients who are renally impaired and those who are obese; results showed good agreement in responses (Additional file 2). However, during Phase 2 it was suggested that the only sub-group warranting separate, specific recommendations were those people with cancer.

The panellists agreed with the participants in Phase 1, that there are multiple individual patient characteristics, such as mobility and concomitant medications, which should all be considered when assessing each individual patient. The panel agreed that the specific patient subgroups discussed (the elderly, patients who are renally impaired and those who are obese) should be managed according to the typical VTE treatment paradigm, and their individual situation (including age, level of renal impairment and body weight) should be considered alongside any other potential risk factors when deciding on the most appropriate DOAC or DOAC dose to prescribe (Table 1).

The Phase 2 panel agreed that the limited consensus observed in Phase 1 on treatment of people with obesity confirmed the uncertainty in the current evidence [14]. Published studies have shown people who are obese are frequently prescribed DOACs [15]. Therefore, the panellists agreed obesity should be considered a risk factor which is important when making treatment decisions, but suggest that this group do not require specific recommendations. The panellists also agreed that all decision making should be in collaboration with the patient, encouraging a shared decision process based on full disclosure of any potential benefits and / or harms of treatments.

**Fig. 5:**
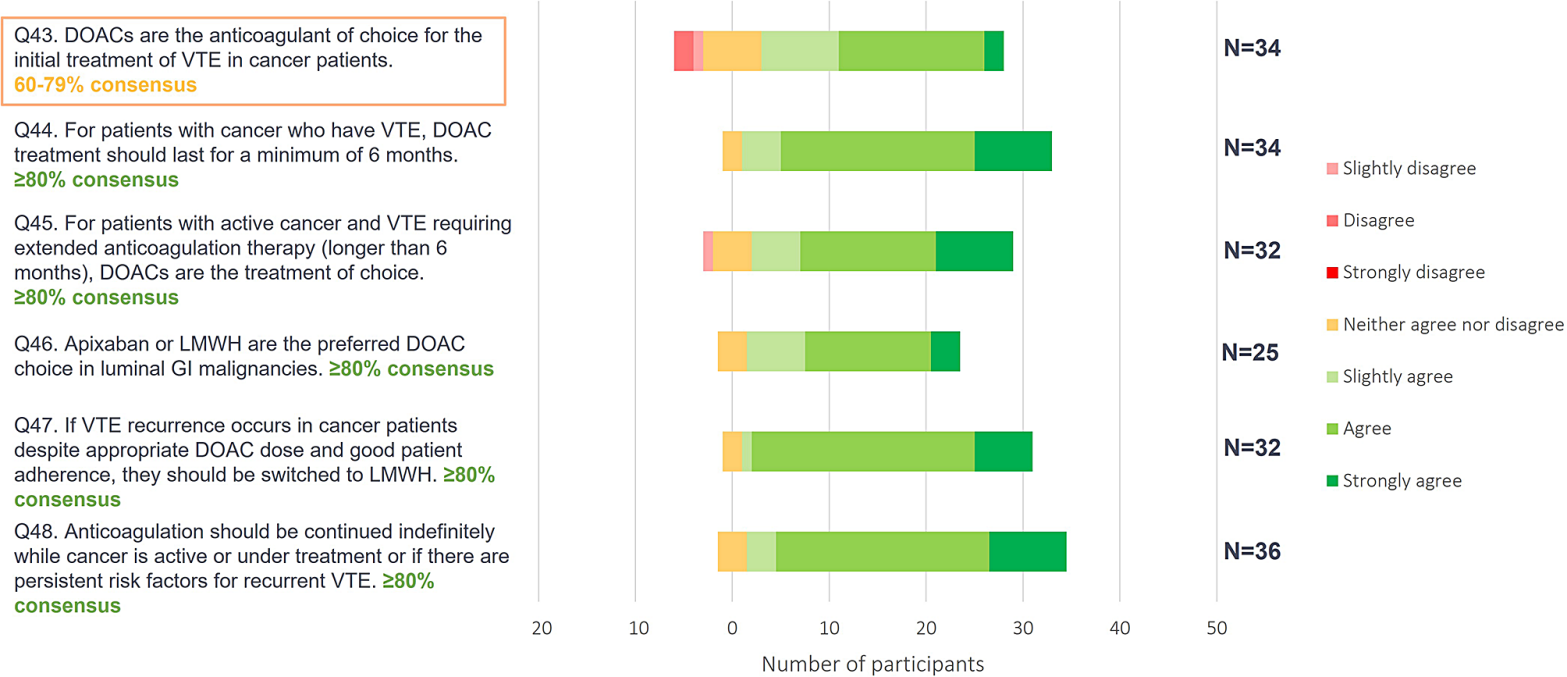
Phase 1 online questionnaire consensus ratings for statements on cancer patients

The statements presented in Phase 1 relating to VTE treatment in patients with cancer received a high level of consensus *(*Fig. 5. *Phase 1 online questionnaire consensus ratings for statements on cancer patients* ), and the Phase 2 panel agreed that DOACs are the treatment of choice for these patients; however, the panellists also noted that patients with gastrointestinal or genitourinary tract cancers, and those aged over 75 years may have a higher bleeding risk, and this should be given additional consideration. DOACs should be used for a minimum of six months in accordance with published guidelines [7]; however, the panel emphasized that active cancer is a provoked, persistent risk factor for consideration when deciding to continue or discontinue with anticoagulant treatment. As patients with cancer require specific consideration, generalist physicians may wish to refer to specialists in antithrombotic therapy when treating their patients with cancer and VTE [16, 17]. It should also be noted that since the conduct of this study the American Society of Clinical Oncology [18] and the European Society for Medical Oncology [19] have updated their guidance documents for treatment of VTE. These recent publications support our recommendation that people with cancer should be given specific consideration.

### Treatment initiation and duration

#### Key recommendations

- *DOACs are the treatment of choice for managing the initial and extended treatment of VTE patients.*
  - *Depending on DOAC prescribed, treatment may require a lead in period of LMWH.*
- *Initial treatment should be stopped after three to six months if the precipitating factor is no longer present, and the clinical course has been uncomplicated.*

Results from Phase 1 demonstrated clear consensus among participants that DOACs should be used as the initial treatment for the management of patients with VTE and for long-term management of VTE. During panel discussion in Phase 2, the importance of determining whether VTE is provoked or unprovoked at presentation was noted, and the general practitioner should evaluate individual patient risk factors which may influence their bleeding risk in making DOAC selection at initiation.

**Fig. 6:**
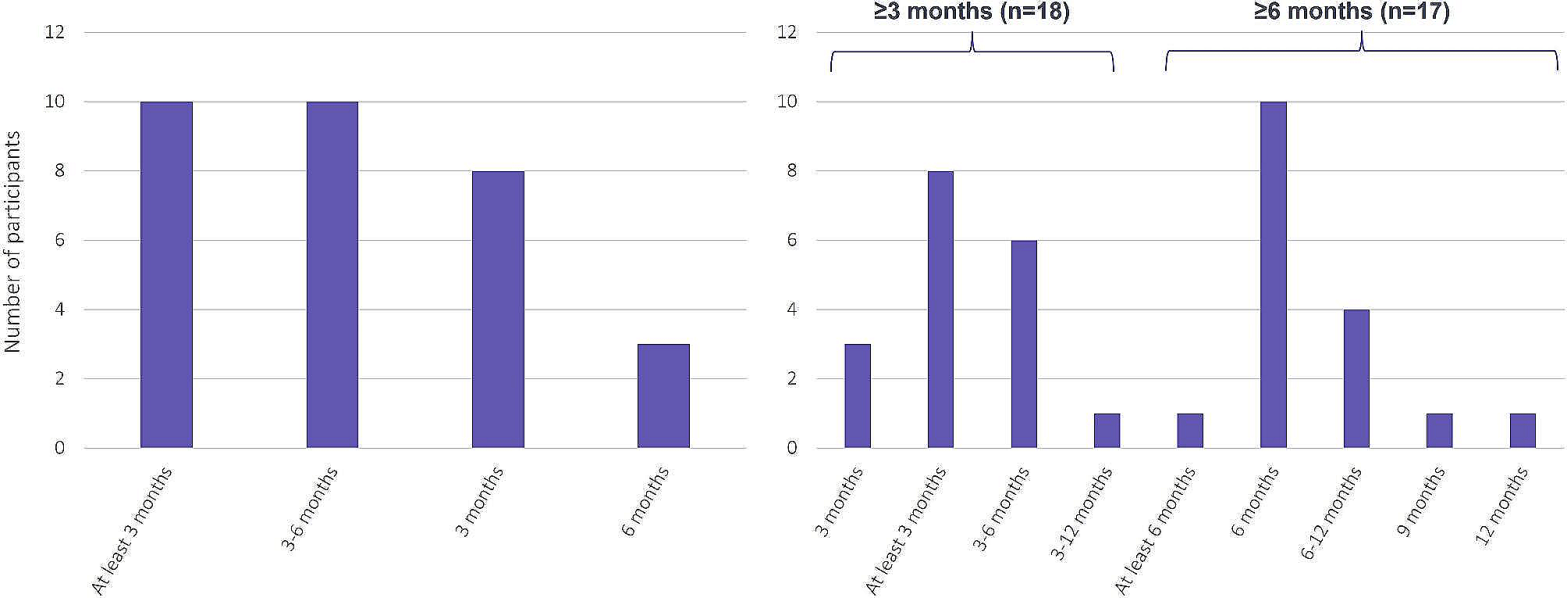
Phase 1 online questionnaire on length of DOAC prescription

In Phase 1 the majority of participants stated that patients with VTE should be reviewed between three and six months (Fig. 6. *Phase 1 online questionnaire on length of DOAC prescription*); although results were mixed, they generally indicated a longer timeframe for those with PE. During Phase 2, the panellists discussed these results, and again noted the length of treatment would depend on whether the VTE was provoked or unprovoked, and duration should be determined according to the individual patient. Panellists agreed with results from Phase 1, that patients should be treated for between three to six months, after which the patient should be reviewed for change in risk factors and risk of bleeding. At the review a decision to continue or cease anticoagulation therapy should be made, and this should be tailored to the individual patient, driven by individual risk factors, patient history and patient choice, such as a particular desire to avoid VTE recurrence, or a preference to discontinue therapy as early as possible.

### Treatment switching

#### Key recommendations

- *If initial DOAC treatment is not well tolerated, consider switching to an alternative DOAC, or refer to a specialist.*
- *For DVT and/or PE patients who present with a new VTE event whilst receiving well controlled VKA treatment, LMWH or DOAC therapy can be prescribed, and consideration for referral to a specialist should be considered.*

There was consensus in results from Phase 1 and Phase 2 that if anticoagulation therapy is still required at the review (three to six months decision point), but the initial DOAC is not well tolerated, generalist physicians should consider switching to another DOAC, or to refer the patient to an antithrombotic specialist. During Phase 2 the panellist agreed that specific recommendations on DOAC switching should not be explicit, and in agreement with findings of Phase 1, no one DOAC is preferable to another.

## Discussion

### Implications for practice

Despite existing guidelines for the treatment of VTE being published, there is still variation in practice within primary care across Europe, considered to be related to uncertainty or confusion over the application of the available guidance in primary care.

The recommendations developed during this formal consensus project align with published guidance [5–10]; however, they aim to provide a simplified summary of the most important considerations. Importantly this research found strong consensus (during Phase 1 and Phase 2) that patients should be considered on an individual basis, and treatment choice should always be a shared decision between the clinician and patient. With this in mind, and to consolidate consensus recommendations a patient pathway for generalist physicians was developed (Fig. 1. *Patient pathway to support generalist physicians in management of VTE*).

### Strengths and limitations

Phase 1 was completed by independent, voluntary (non-reimbursed) participants. We should acknowledge that only 40 participants fully completed the questionnaire, and that if the number of recruited participants had been different, the levels of consensus observed may have altered.

The participants in both Phase 1 and Phase 2 represented a geographical diverse group, supporting generalizability of these recommendations across Europe. Two participants who completed the questionnaire were from outside of Europe (Israel and the UAE); however, we included their responses to gain a broader reflection of practice. The majority of Phase 1 participants were from general practice, without any specialist cardiovascular training, and therefore representative of the target audience that these consensus recommendations have been developed to support. However, it should also be noted, that a proportion of included participants were considered specialists, and are not considered the target audience.

Despite these strengths, some limitations should be considered. Firstly, selection bias towards those individuals with interest in cardiovascular disease completing the online questionnaire cannot be ruled out; however, the majority of individuals were from within primary care and therefore considered generalists. Additionally, we did not request any information on the participants’ conflicts of interest; therefore we cannot rule out any bias in the responses provided.

Secondly, by developing simple consensus recommendations, not all nuances of management can be incorporated, and some aspects of care may not be covered. The final consensus statements were not validated by all of those participating in Phase 1; however, three members of the panel in Phase 2 completed Phase 1, giving partial validation; additionally, there was strong agreement by the panellists in Phase 2 with the responses given in Phase 1, providing confidence in the consensus recommendations developed. Indeed, the project was designed to balance breadth and depth in expertise, by having a wider group of participants in Phase 1 and a smaller expert group for deep discussion to develop practical guidance during Phase 2.

## Conclusion

This formal consensus exercise gathered and consolidated opinions from generalist community physicians from across Europe. The findings demonstrate an appreciation of best practices among those managing VTE in primary care, but highlights challenges experienced in clinical practice.

The patient pathway developed, and consensus recommendations provided, aim to highlight key considerations for the generalist physician to facilitate decision making and to aid optimal VTE treatment.

### Electronic supplementary material

Below is the link to the electronic supplementary material.

Supplementary Material 1

Supplementary Material 2

## Data Availability

All data generated and/or analysed during the current study are not publicly available due to privacy reasons, but are available from the corresponding author on reasonable request.

## Abbreviations

DVT: Deep vein thrombosis
DOAC: Direct oral anticoagulant
LMWH: Low molecular weight heparin
PE: Pulmonary embolism
PCCS: Primary Care Cardiovascular Society
VKA: Vitamin K antagonist
VTE: Venous thromboembolism
