# Supplementary material for: European expert consensus recommendations on the primary care use of direct oral anticoagulants in patients with venous thromboembolism
Source: BMC Prim Care. 2024 Mar 18;25:90. doi: 10.1186/s12875-024-02314-7 (PMC10946109; doi:10.1186/s12875-024-02314-7)
Supplement: Supplementary file 1 — Supplementary Material 1 [file 12875_2024_2314_MOESM1_ESM.docx]

**Supplementary materials**

**Supplementary section 1**

**Key guidance documents to support development of statements for Phase 1 Delphi**

*General management*

- **American Society of Hematology (ASH 2020)**

Guidelines for management of venous thromboembolism: treatment of deep vein thrombosis and pulmonary embolism

- **The National Institute for Health and Care Excellence (NICE 2020)**

Venous thromboembolic diseases: diagnosis, management and thrombophilia testing

- **The American College of Chest Physicians (CHEST 2021)**

Antithrombotic Therapy for VTE Disease: Second Update of the CHEST Guideline and Expert Panel Report – Executive Summary

- **European Society of Cardiology (ESC 2019)**

Guidelines for the diagnosis and management of acute pulmonary embolism developed in collaboration with the European Respiratory Society (ERS)

*Elderly populations*

- **Review – Roberti *et al.* (2021)**

Direct Oral Anticoagulants: From Randomized Clinical Trials to Real-World Clinical Practice. Frontiers in pharmacology, 12, 684638.

- **Review -Gross & Chan (2021).** Thromboembolism in older adults. Frontiers in medicine, 7, 470016
- **Review – Li *el al.* (2016).**

Use of Direct Oral Anticoagulants in Special Populations. Hematology/oncology clinics of North America, 30(5), 1053–1071.

- **Review – Boey & Gallus (2016)**

Drug Treatment of Venous Thromboembolism in the Elderly

*Renally impaired populations*

- **The National Institute for Health and Care Excellence (NICE 2020)**

Venous thromboembolic diseases: diagnosis, management and thrombophilia testing

- **Medicines & Healthcare products Regulatory Agency guidance (2020)**

Drug safety update: Direct-acting oral anticoagulants (DOACs): reminder of bleeding risk, including availability of reversal agents

- **Review – Li *el al.* (2016)**

Use of Direct Oral Anticoagulants in Special Populations. Hematology/oncology clinics of North America, 30(5), 1053–1071.

*Obese populations*

- **International Society on Thrombosis and Haemostasis (ISTH 2021)**

Use of direct oral anticoagulants in patients with obesity for treatment and prevention of venous thromboembolism: Updated communication from the ISTH SSC Subcommittee on Control of Anticoagulation

- **Review - Sebaaly & Kelley. (2020).**

Direct Oral Anticoagulants in Obesity: An Updated Literature Review. The Annals of pharmacotherapy, 54(11), 1144–1158.

*Patients with cancer*

- **American Society of Hematology (ASH 2021)**

Guidelines for management of venous thromboembolism: prevention and treatment in patients with cancer

- **The National Institute for Health and Care Excellence (NICE 2020)**

Venous thromboembolic diseases: diagnosis, management and thrombophilia testing

- **The American College of Chest Physicians (CHEST 2021)**

Antithrombotic Therapy for VTE Disease: Second Update of the CHEST Guideline and Expert Panel Report – Executive Summary

- **International Society on Thrombosis and Haemostasis (ISTH 2019)**

International clinical practice guidelines for the treatment and prophylaxis of venous thromboembolism in patients with cancer

- **Spanish Society of Medical Oncology (SSMO 2019)**

Clinical guideline of venous thromboembolism (VTE) and cancer

- **National Comprehensive Cancer Network (NCCN 2020)**

Cancer-associated venous thromboembolic disease

- **American Society of Clinical Oncology clinical guidelines (ASCO 2019)**

Venous Thromboembolism Prophylaxis and Treatment in Patients With Cancer: ASCO Clinical Practice Guideline Update

- **European Society for Medical Oncology (2011)**

Management of venous thromboembolism (VTE) in cancer patients: ESMO Clinical Practice Guidelines
