# Supplementary material for: European expert consensus recommendations on the primary care use of direct oral anticoagulants in patients with venous thromboembolism
Source: BMC Prim Care. 2024 Mar 18;25:90. doi: 10.1186/s12875-024-02314-7 (PMC10946109; doi:10.1186/s12875-024-02314-7)

**Supplementary materials 2**

**Phase 1 Online questionnaire results**

Q1 to Q3 – Confidential responses relating to the participants in Phase 1 are not provided.

Q4. In which clinical setting do you work, please tick all that apply:

General Practice = 29, Primary care specialist = 11, Secondary care = 6, specialist centre = 16.

Q5. Please estimate the total number of patients managed in your clinic during a typical year.

Range 10-27, 000. Average 4,500

Q6. Please estimate what proportion of these patients have VTE.

Range 1=100%. Average 17%

Q7. Please estimate the proposition of these patients with VTE in whom DOACs are prescribed

Range 0-100%. Average 60%

Supplementary Figure 1. Phase 1 online questionnaire consensus ratings on the current VTE treatment landscape


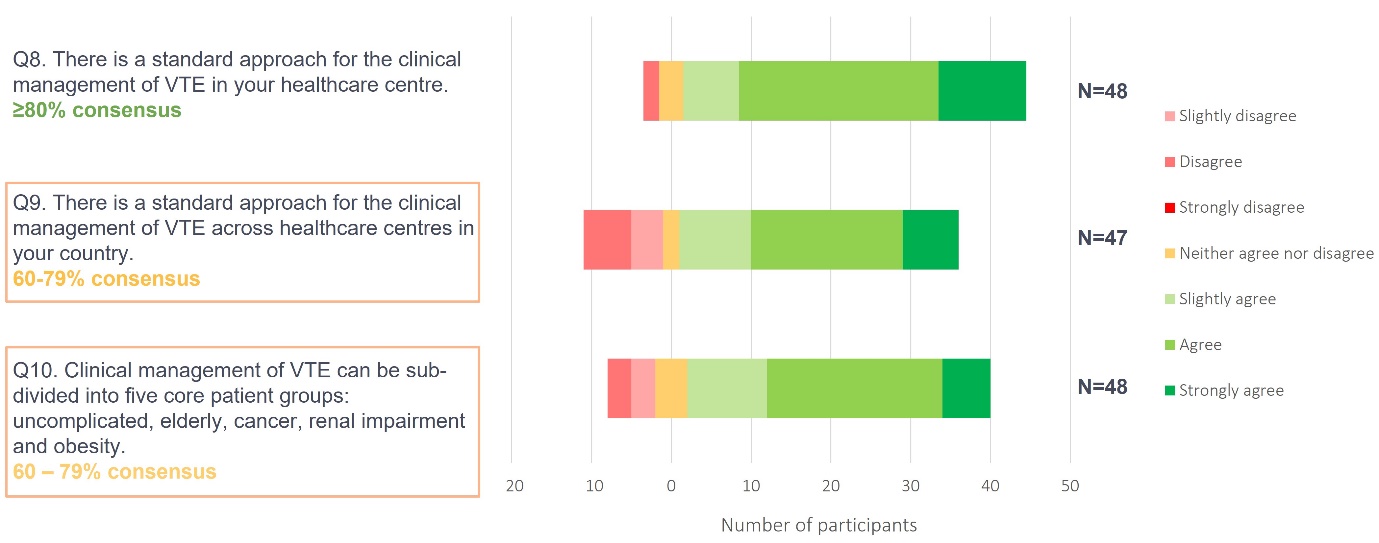


Supplementary Figure 2. Phase 1 online questionnaire open question, ‘What are the key patient risk factors that define clinical management of VTE?’


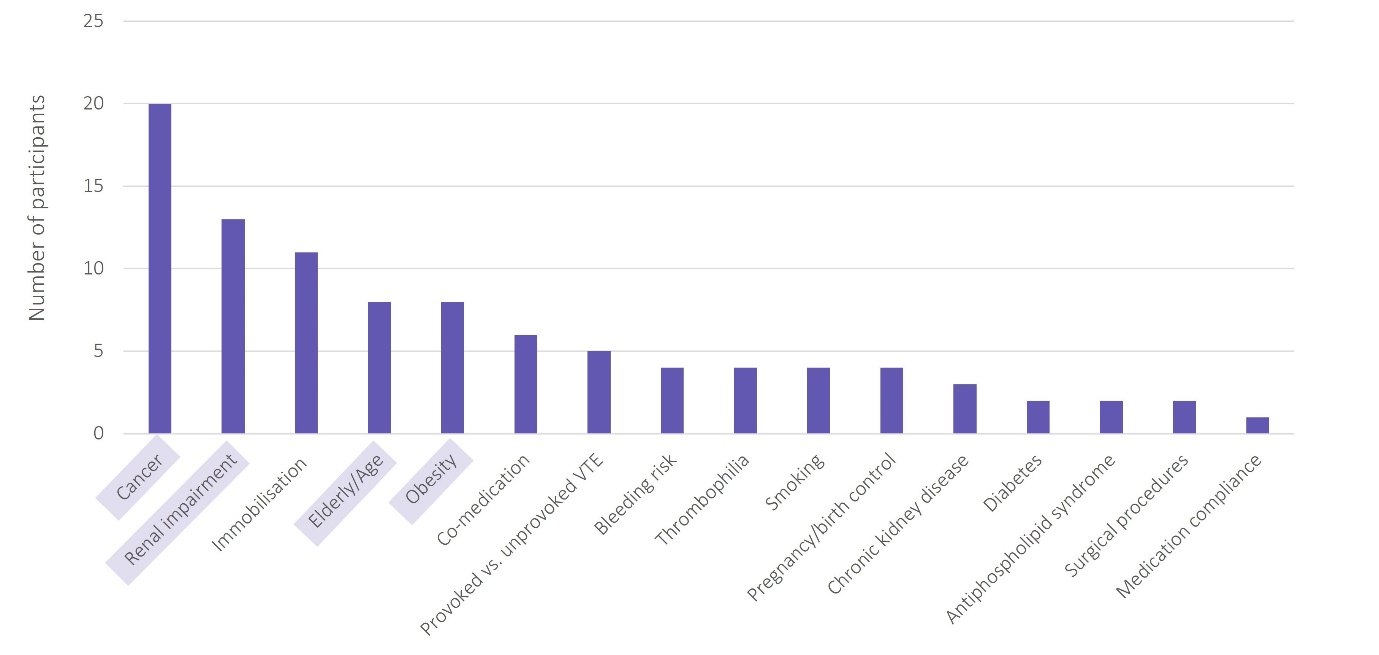


Supplementary Figure 3. Phase 1 online questionnaire consensus ratings for DOACs versus other anticoagulants


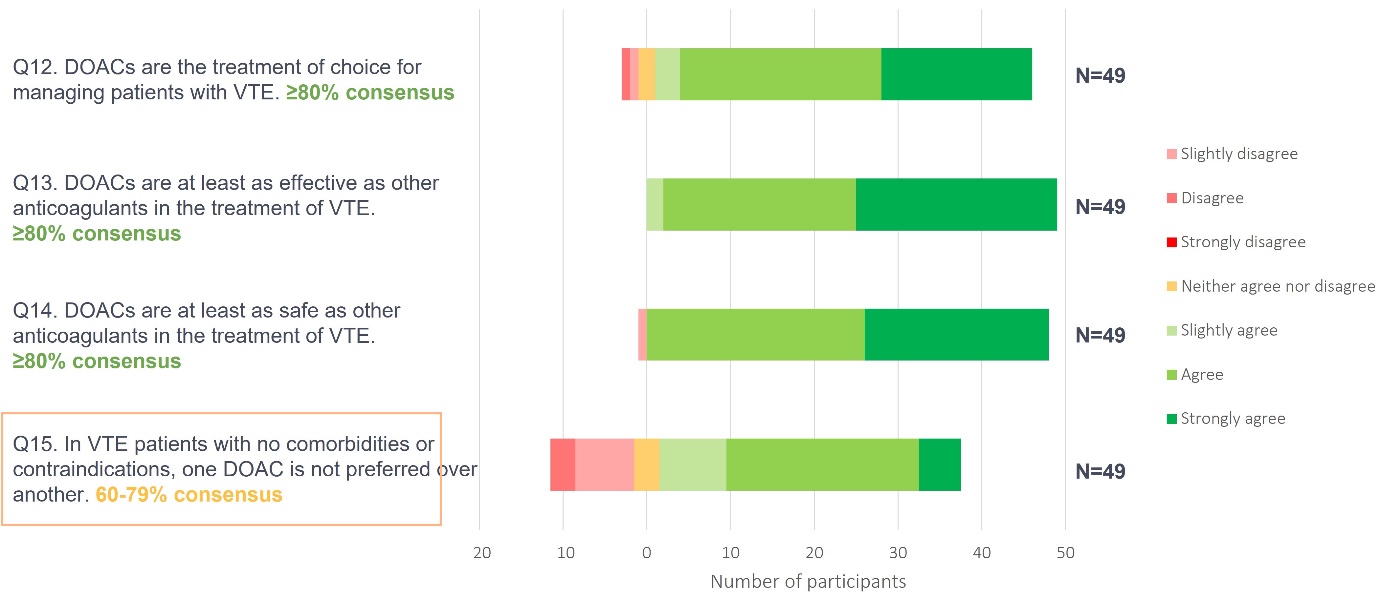


Supplementary Figure 4. Phase 1 online questionnaire consensus ratings for DOACs versus other anticoagulants


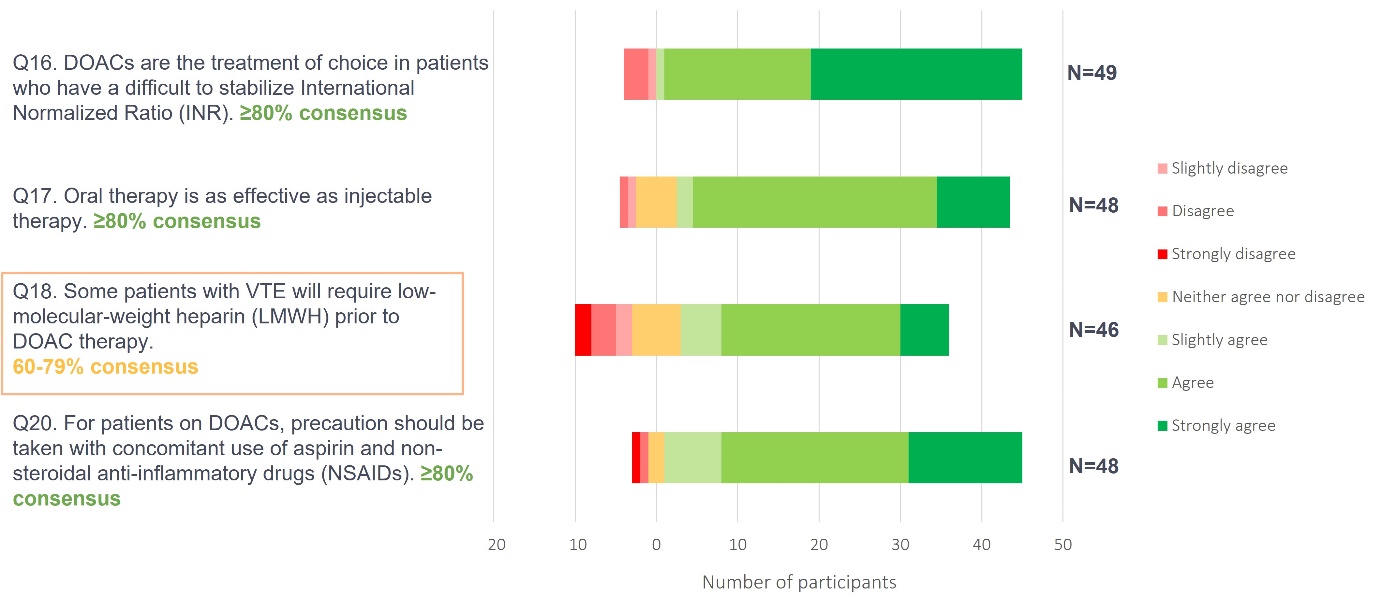


Supplementary Figure 5. Phase 1 online questionnaire consensus ratings for managing the initial treatment of VTE patients


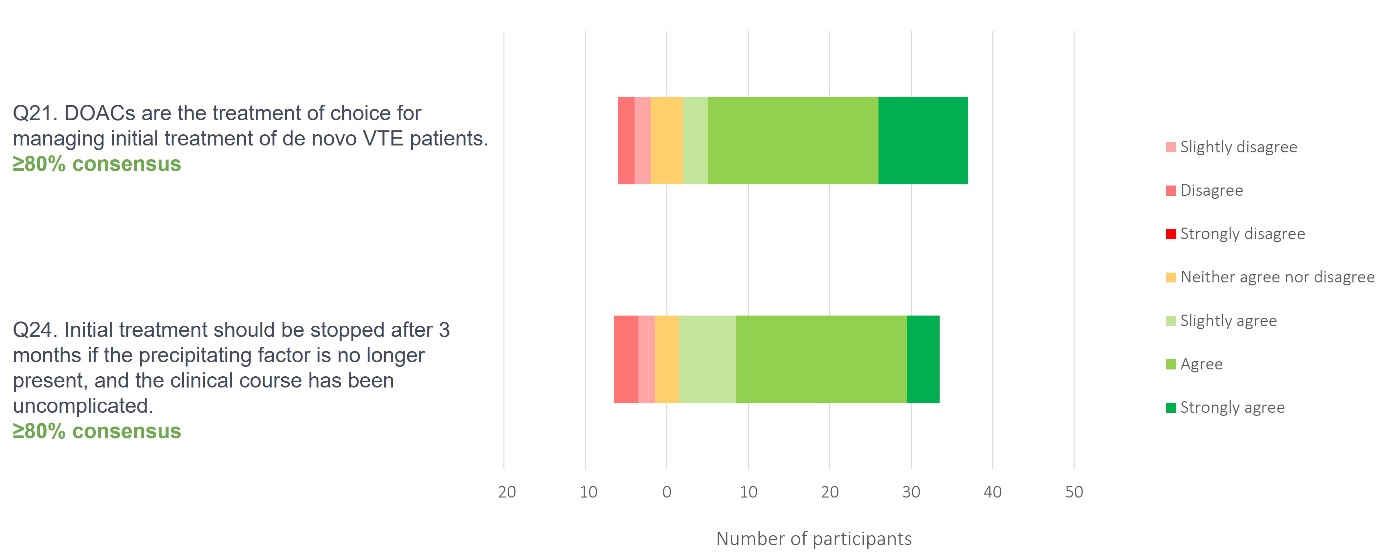


Supplementary Figure 6. Phase 1 online questionnaire consensus ratings for managing the long-term treatment of VTE patients


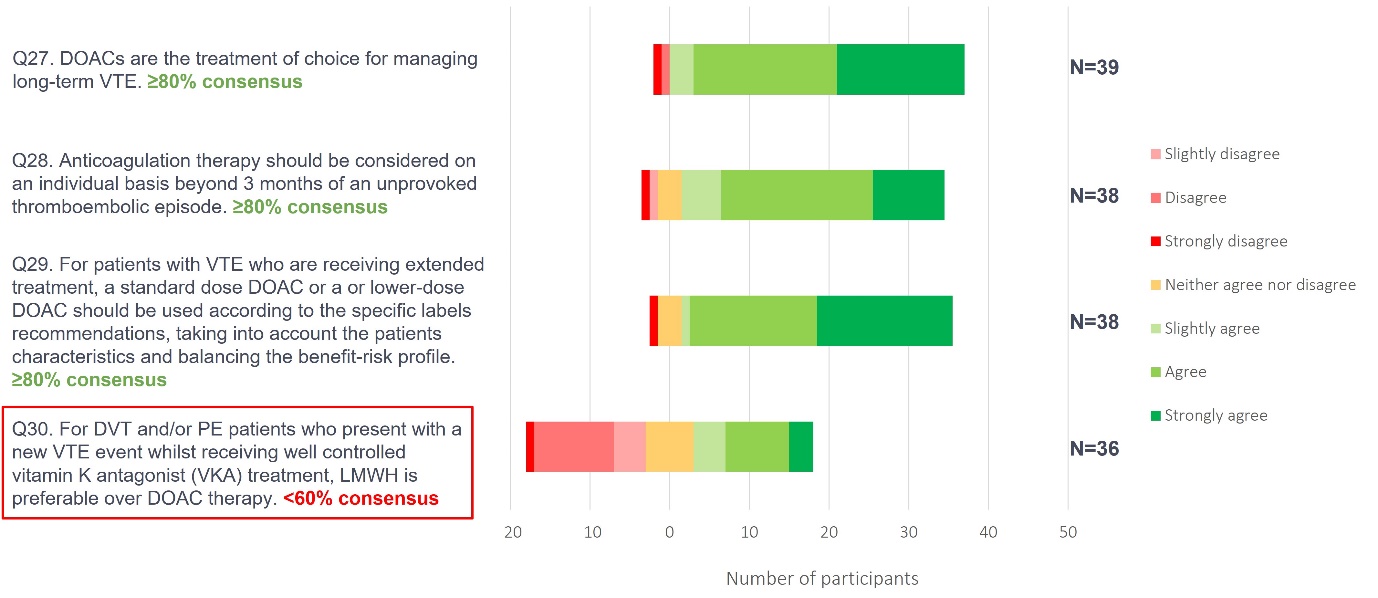


Supplementary Figure 7. Phase 1 online questionnaire open questions, ‘For how long would you prescribe DOACs to de novo DVT and PE patients?’


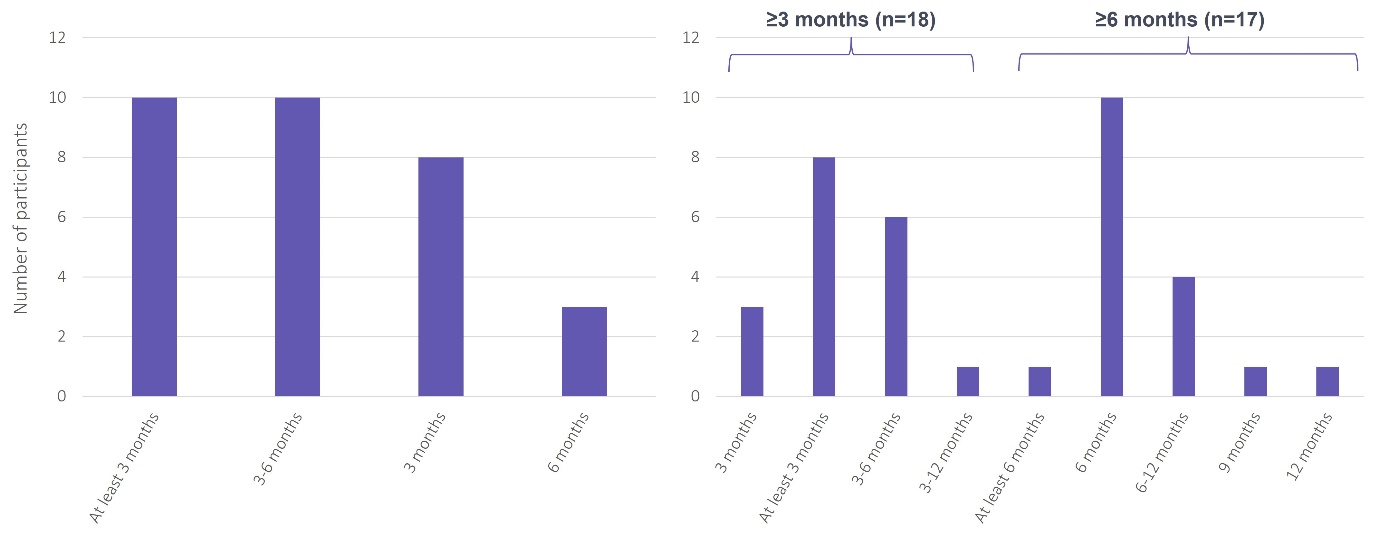


Supplementary Figure 8. Phase 1 online questionnaire open question, ‘If the initial DOAC prescribed (please state) is not well tolerated, what would you switch treatment to?’


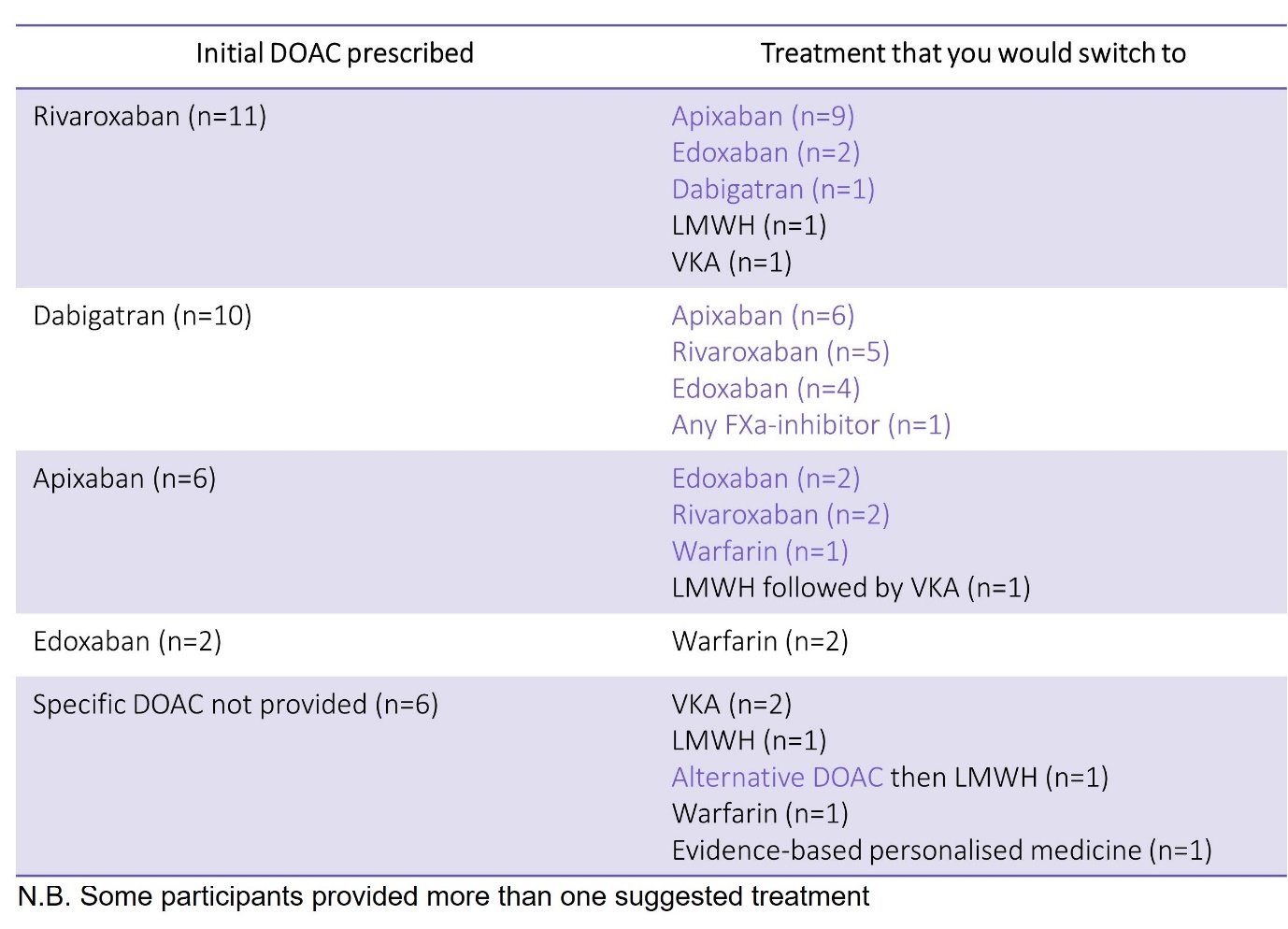


Supplementary Figure 9. Phase 1 online questionnaire open question, ‘What would be your DOAC of choice (if at all) for a VTE patient with a history of GI bleeding or at risk for GI bleeding?’


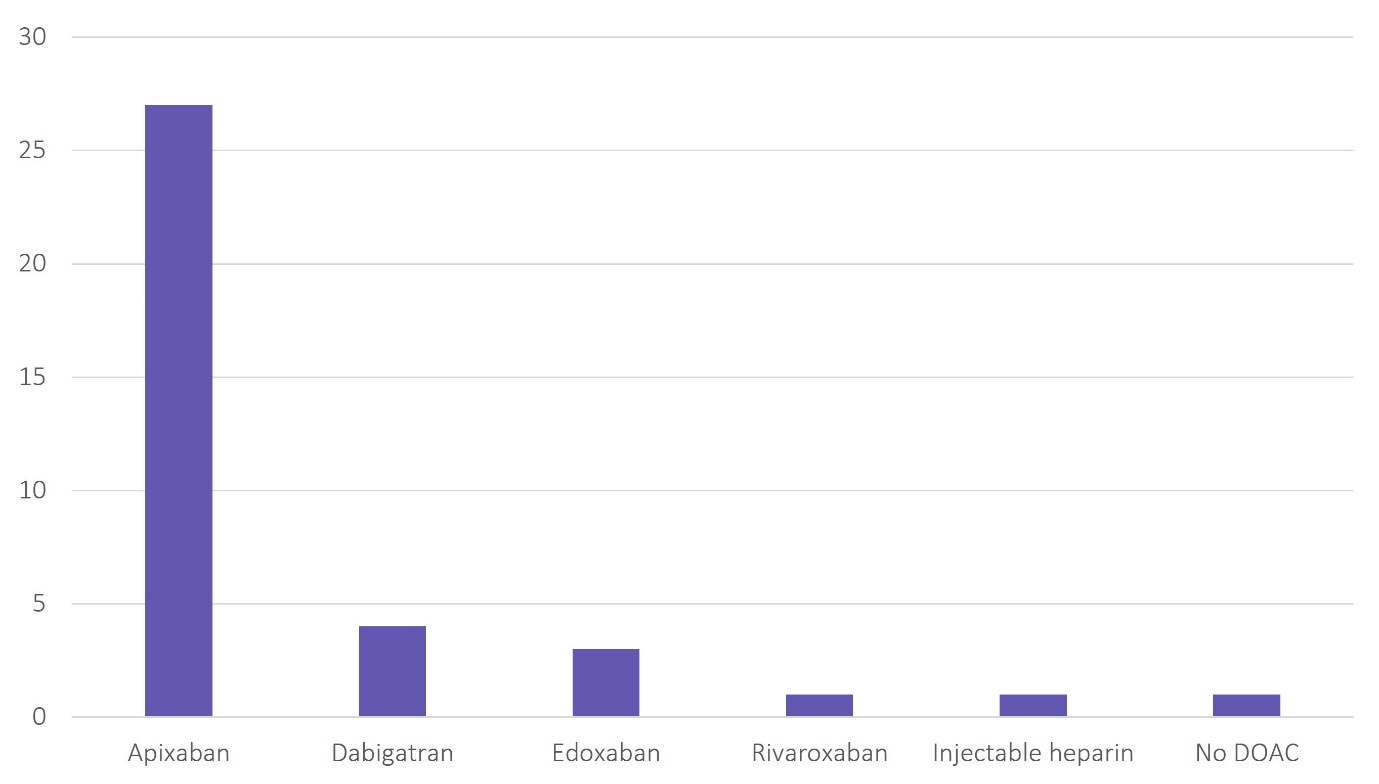


Supplementary Figure 10. Phase 1 online questionnaire consensus ratings for statements on obese patients


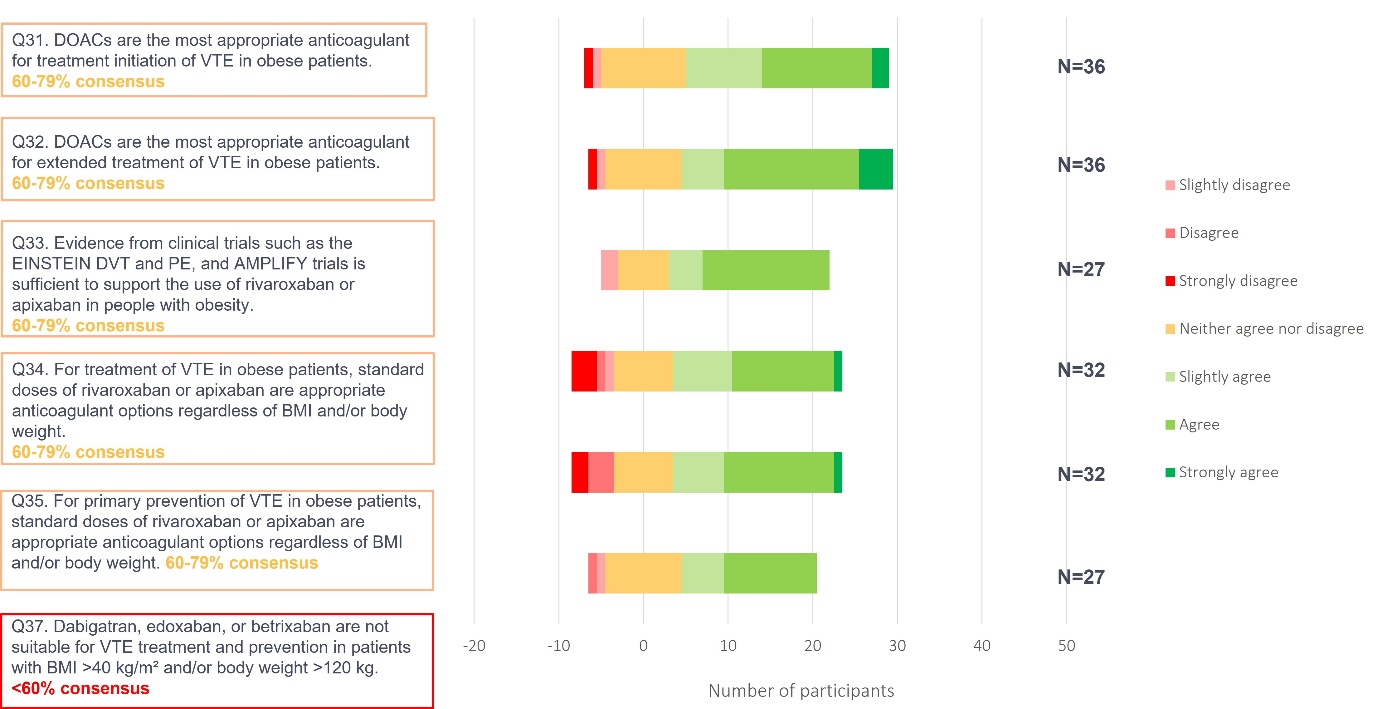


Supplementary Figure 11. Phase 1 online questionnaire consensus ratings for statements on elderly patients


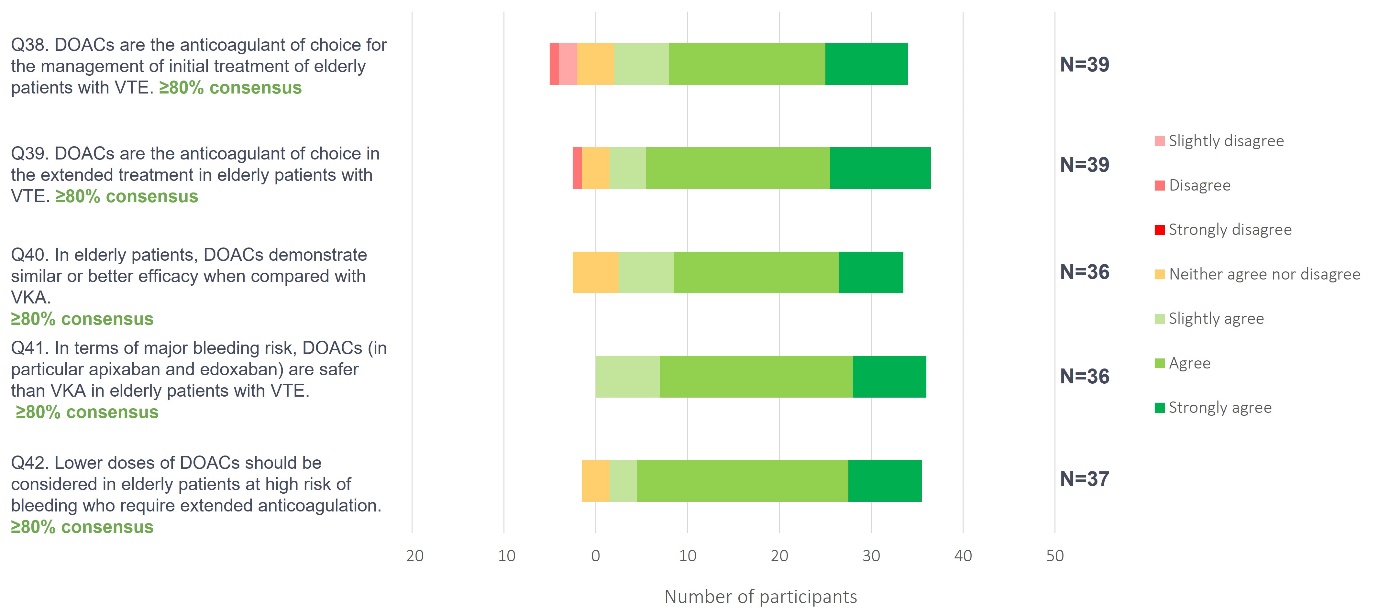


Supplementary Figure 12. Phase 1 online questionnaire consensus ratings for statements on cancer patients

*
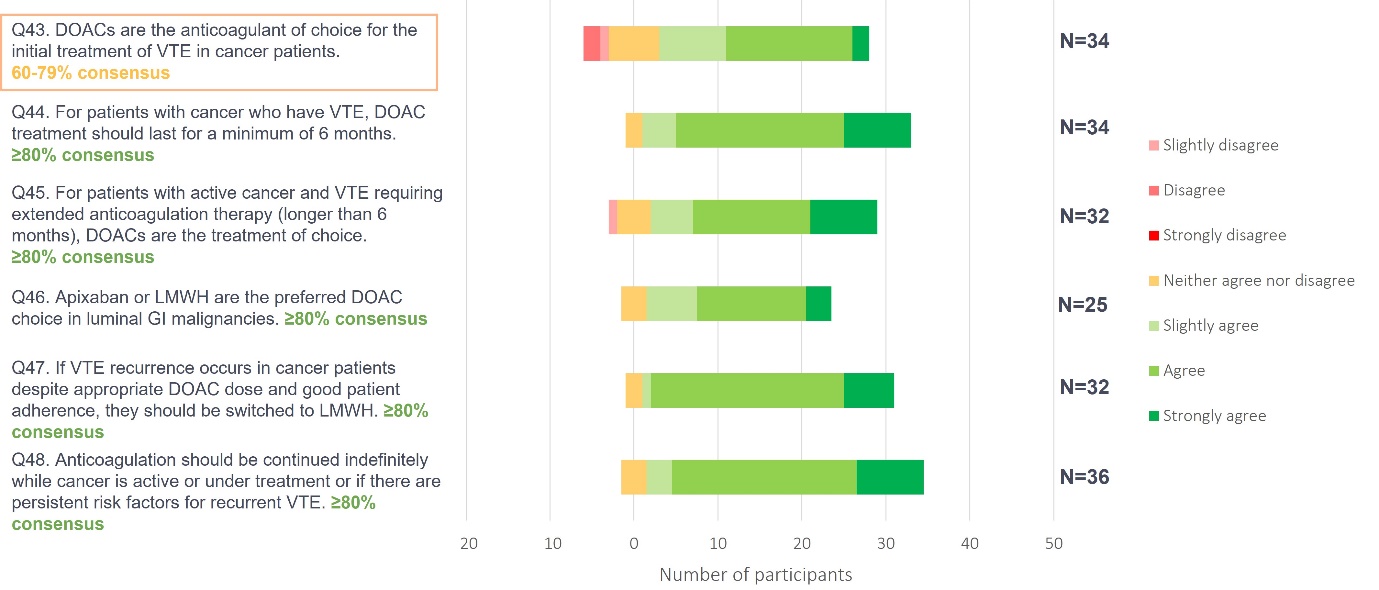
*

Supplementary Figure 13. Phase 1 online questionnaire consensus ratings for statements on renally impaired patients


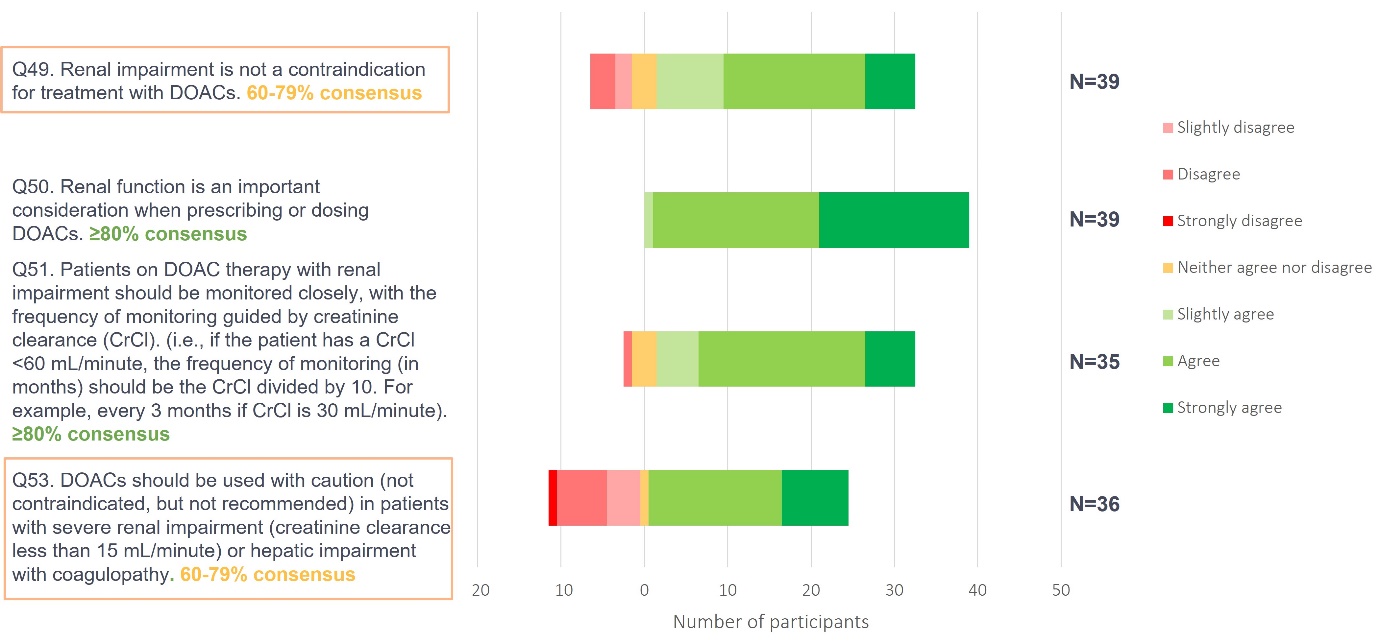

Supplement: Supplementary file 2 — Supplementary Material 2 [file 12875_2024_2314_MOESM2_ESM.docx]
